# Pathological Brain Detection Using Weiner Filtering, 2D-Discrete Wavelet Transform, Probabilistic PCA, and Random Subspace Ensemble Classifier

**DOI:** 10.1155/2017/4205141

**Published:** 2017-10-03

**Authors:** Debesh Jha, Ji-In Kim, Moo-Rak Choi, Goo-Rak Kwon

**Affiliations:** ^1^Department of Information and Communication Engineering, Chosun University, 309 Pilmun-Daero, Dong-Gu, Gwangju 61452, Republic of Korea; ^2^School of Electrical Engineering, Korea University, 145 Anam-ro, Sungbuk-gu, Seoul 02841, Republic of Korea

## Abstract

Accurate diagnosis of pathological brain images is important for patient care, particularly in the early phase of the disease. Although numerous studies have used machine-learning techniques for the computer-aided diagnosis (CAD) of pathological brain, previous methods encountered challenges in terms of the diagnostic efficiency owing to deficiencies in the choice of proper filtering techniques, neuroimaging biomarkers, and limited learning models. Magnetic resonance imaging (MRI) is capable of providing enhanced information regarding the soft tissues, and therefore MR images are included in the proposed approach. In this study, we propose a new model that includes Wiener filtering for noise reduction, 2D-discrete wavelet transform (2D-DWT) for feature extraction, probabilistic principal component analysis (PPCA) for dimensionality reduction, and a random subspace ensemble (RSE) classifier along with the *K*-nearest neighbors (KNN) algorithm as a base classifier to classify brain images as pathological or normal ones. The proposed methods provide a significant improvement in classification results when compared to other studies. Based on 5 × 5 cross-validation (CV), the proposed method outperforms 21 state-of-the-art algorithms in terms of classification accuracy, sensitivity, and specificity for all four datasets used in the study.

## 1. Introduction

Magnetic resonance imaging (MRI) of the brain provides comprehensive diagnostic information for diagnosis [1]. It is essential because it is noninvasive and safe and yields a higher resolution that cannot be obtained by other techniques. MRI is mainly utilized to diagnose different types of disorders such as strokes, tumors, bleeding, injury, blood-vessel diseases or infections, and multiple sclerosis (MS). The early diagnosis of pathological brain disease and its prodromal stage are critical and can decrease or halt the progression of the disease [2]. Therefore, the classification of normal/pathological brain status from MRIs is essential in clinical medicine as it focuses on soft tissue anatomy and generates a large and detailed dataset about the subject's brain. However, the use of a large database makes manual interpretation of the brain images tedious, time consuming, and costly. The major drawback of the manual approach is its irreducibility. Therefore, there is a need for automated image analysis tools such as computer-aided diagnosis (CAD) systems [3].

Considerable research has been carried out to develop automatic tools for the classification of MR images to distinguish between normal and pathological brains. El-Dahshan et al. [4] utilized a three-level discrete wavelet transform, accompanied by principal component analysis (PCA), to decrease features. A good success rate was obtained by using feedforward backpropagation neural networks (BPNNs) and the *K*-nearest neighbor (KNN). Zhang and Wu [5] recommended the application of a kernel support vector machine (KSVM) and presented three new kernels: homogenous polynomial, inhomogeneous polynomial, and Gaussian radial basis for distinguishing between normal and abnormal images. Patnaik et al. [6] employed DWT to obtain the approximation coefficients. Later, a support vector machine (SVM) was utilized to perform the classification. Zhang et al. [7] recommended a training feedforward neural network (FNN) with a unique scaled conjugate gradient (SCG) technique. Kundu et al. [8] proposed combining the Ripplet transform (RT) for feature extraction, PCA for dimensionality reduction, and the least-square SVM (LS-SVM) for classification, and the 5 × 5 stratified cross-validation (SCV) offered high classification accuracies. El-Dahshan et al. [9] utilized the feedback pulse-coupled neural network for the preprocessing of MR images, the DWT for feature extraction, PCA for features reduction, and the FBPNN for the classification of pathological and normal brains. Damodharan and Raghavan [10] used wavelet entropy as the feature space, and they then used the traditional naïve-Bayes classifier classification method. Wang et al. [11] utilized the stationary wavelet transform (SWT) to substitute for DWT. Likewise, they proposed a hybridization of particle swarm optimization (PSO) and the artificial bee colony (HPA) method to obtain the optimal weights and biases of FNN. Nazir et al. [12] applied denoising at the beginning, and they achieved an overall classification accuracy of 91.8%. Harikumar and Vinoth Kumar [13] used wavelet-energy and SVM. Padma and Sukanesh [14] used the combined wavelet statistical feature to segment and classify Alzheimer's disease (AD) as well as benign and malignant tumor slices. Zhang et al. [15] utilized Hu moment invariants (HMI) and generalized eigenvalue proximal SVM (GEPSVM) for the detection of pathological brain in MRI scanning and obtained an accuracy of 98.89%, sensitivity of 99.29%, and specificity of 92.00%. Later on, Zhang et al. [16] used multilayer perceptron (MLP) for classification, where two pruning techniques like dynamic pruning (DP) and Bayesian detection boundaries (BDB were used to find the optimal hidden neurons and an adaptive real coded BBO (ARCBBO) method was implemented to determine the optimal weights and obtained an accuracy of 98.12% and 98.24%, respectively. Nayak et al. [17] used 2D-DWT, PCA, and Adaboost algorithm with random forest as its base classifier and obtained an accuracy of 98.44% for classification of pathological brain MR image with Dataset-255. Later on, Nayak et al. [18] utilized two-dimensional stationary wavelet transform (SWT), symmetric uncertainty ranking (SUR) filter, and Adaboost with SVM classifier for the detection of pathological brain MR images and obtained an accuracy of 98.43% with Dataset-255. Wang et al. [19] employed Pseudo Zernike moment and linear regression classifier for classification of Alzheimer's disease and yielded an accuracy of 97.51%, sensitivity of 96.71%, and specificity of 97.73%. Alam et al. [20] utilized dual-tree complex wavelet transform (DTCWT), principal component analysis (PCA), and twin support vector machine (TSVM) for the detection of Alzheimer's disease classification and obtained an accuracy of 95.46 ± 1.26.

Scholars have proposed different methods to extract features for the pathological brain disease [21]. After analyzing the above methods, we found that all of the methods achieved promising results which indicated that 2D-DWT is effective in feature extraction for pathological brain detection. However, there are two problems. (1) Most of them utilize traditional PCA for feature extraction which is computational-intensive for large datasets with a higher dimensions. (2) The classification performance can be further improved, because the feature vector contains excessive features, which required more memory and increased computational complexity. Moreover, it required too much time to train the classifiers.

To address the above-mentioned problems, we proposed a new pathological brain detection system based on brain MR images which has the potential improvements over the other schemes. Weiner filter is used for the preprocessing of the images. The proposed method uses 2D DWT for the extraction of features because of its ability to analyze images at different scales. PPCA is used in place of PCA for the reduction of features which has the advantages of computing the efficient dimension reduction in terms of the distribution of latent variables, maximum-likelihood estimates, probability model, dealing with the missing data, and a combination of multiple PCA as probabilistic mixture. A relatively new classifier known as random subspace ensemble (RSE) classifier is employed which has the advantage of low computational burden over the traditional classifiers. Hence, the novelty of the proposed method lies in the application of PPCA features and RSE classifier.

The article is organized as follows: Section 2 presents details about the materials and methods.  Section 3 describes the experimental results, evaluation procedure, and discussions. Finally, Section 4 presents the conclusion and future research.

## 2. Materials and Methods

### 2.1. Materials

At present, there are four benchmark datasets (DS) as DS-66, DS-90, DS-160, and DS-255, of different sizes of 66, 90, 160, and 255 images, respectively. All the datasets (DS) contain axial, T2-weighted, 256 × 256-pixel MR images downloaded from medical school of Harvard University (Boston, MA, USA) (URL: http://www.med.harvard.edu/aablib/home.html) website. T2-weighted images are selected as input image because T2-weighted (spin-spin) relaxation gives better image contrast that is helpful to show different anatomical structure clearly. Also, they are better in detecting lesions than T1 weighted images.

We selected five slices from each subject. The selection criterion is that, for healthy subjects, these slices were selected at random. For pathological subjects, the slices should contain the lesions by confirmation of these radiologists with ten years of experiences. A sample of diseased slices is shown in Figure 2. In this investigation, all diseases are treated as pathological, and our task is a binary classification problem, that is, to distinguish pathological brain from healthy brains. Here, the whole brain is considered as the input image. We did not select local characteristics like point and edge, and we extract global image characteristics that are further learned by the new cascade model. Let us keep in mind that our procedure is different from the way neuroradiologists do. They usually take the local features and compare with standard template to check whether focuses exist, such as shrink, expansion, bleeding, and inflammation. While our technique is like AlphaGO, the computer researcher gives the machine sufficient data, and then the machine can learn how to make classification naturally. Including patients' information (age, gender, handedness, memory test, education, etc.) can add additional information and thus may assist us to improve the classification performance. Nevertheless, this new model proposed in our research is only dependent on the imaging data. Besides, the imaging data from the website does not contain the subjects' information.

The cost of predicting pathological to normal types is severe, because the subjects may be told that she/he is normal and thus avoids the mild symptoms displayed. The treatments of patients may be postponed. Nevertheless, the cost of misclassification of healthy to pathological types is low, since correct treatment can be given by other diagnosis means. The cost-sensitivity (CS) problem was resolved by changing the class distribution at the beginning state, since original data was accessible. That means we purposely picked up more pathological brains than healthy ones into the dataset, with the goal of making the classifier biased to pathological brains, to solve the CS problem. The overfitting problem was supervised by cross-validation technique.

In our experiment, DS-66 and DS-160 are extensively employed for brain MR image classifications that consist of normal brain images as well as abnormal brain images from seven types of diseases, namely, glioma, meningioma, Alzheimer's disease, Alzheimer's disease plus visual agnosia, Pick's disease, sarcoma, and Huntington's disease. DS-90 contains MR brain images of a healthy brain, AIDS dementia, Alzheimer's disease plus visual agnosia, Alzheimer's disease, cerebral calcinosis, cerebral toxoplasmosis, Creutzfeldt-Jakob disease, glioma, herpes encephalitis, Huntington's disease, Lyme encephalopathy, meningioma, metastatic adenocarcinoma, metastatic bronchogenic carcinoma, motor neuron disease, MS, Pick's disease, and sarcoma.

The third dataset, DS-255, includes images of four new types of diseases embedded with the above seven types of diseased images and normal brain images. The four additional diseases are chronic subdural hematoma, cerebral toxoplasmosis, herpes encephalitis, and MS.

### 2.2. Proposed Methodology

**Figure 1:**
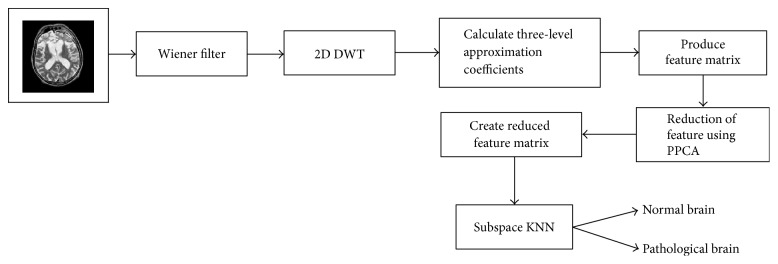
Block diagram of the proposed system.

**Figure 2:**
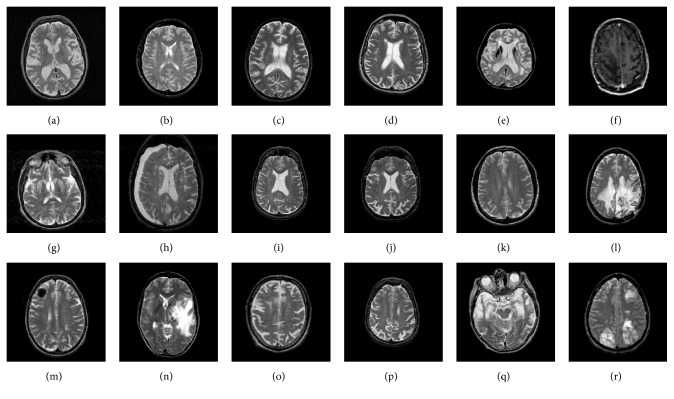
Brain MR images: (a) healthy brain; (b) AIDS dementia; (c) Alzheimer's disease plus visual agnosia; (d) Alzheimer's disease; (e) cerebral calcinosis; (f) cerebral toxoplasmosis; (g) Creutzfeldt-Jakob disease; (h) glioma, (i) herpes encephalitis; (j) Huntington's disease; (k) Lyme encephalopathy; (l) meningioma; (m) metastatic adenocarcinoma; (n) metastatic bronchogenic carcinoma; (o) motor neuron disease; (p) multiple sclerosis; (q) Pick's disease; and (r) sarcoma.

The proposed method comprises four vital stages, namely, image preprocessing, feature extraction using 2D-DWT, feature reduction utilizing PPCA, and classification using the RSE classifier. In order to enhance the quality of the MR images, Wiener filter is employed, followed by the extraction of approximation coefficients from MR images utilizing a 2D-DWT with three-level decomposition. Then, we saved these obtained features as our primary features. Thereafter, then we employ PPCA for obtaining uncorrelated discriminant set of features. Finally, we classified the reduced features using the RSE classifier with KNN as a base classifier. The complete block diagram of the proposed system is shown in Figure 1. A brief description about all these four stages is shown below.

#### 2.2.1. Preprocessing Using Wiener Filter

The gif images were downloaded individually from the website of the Harvard Medical School. Then, each of the gif images was converted into JPG format manually. The images were in RGB format, and they were then converted into grayscale intensity images. Next, the intensity image is converted to double precision. Acquired brain MR images require preprocessing to improve the quality, enabling us to obtain better features. In our study, we used the popular Wiener filter method.

The Wiener filter is used to replace the finite impulse response (FIR) filter in order to decrease noise in signals [22]. When an image is blurred by a familiar low-pass filter (LPF), we can recover the image by inverse filtering. However, inverse filtering is extremely sensitive to additive noise. Wiener filtering accomplishes an optimal trade-off between inverse filtering and noise smoothing in that it eliminates the additive noise and inverts the blurring simultaneously. In addition, it reduces the overall mean-square error during the course of inverse filtering plus noise smoothing. The Wiener filtering method generates a linear approximation of the original image and is based on the stochastic framework. The orthogonality principle indicates that the Wiener filter in the Fourier domain can be articulated as follows:

(1) $$\begin{array}{c} W\left(\;f_{1},f_{2}\;\right)=\frac{H^{*}\left(f_{1},f_{2}\right)S_{xx}\left(f_{1},f_{2}\right)}{{\left\|H\left(f_{1},f_{2}\right)\right\|}^{2}S_{xx}+S_{nn}\left(f_{1},f_{2}\right)}. \end{array}$$

Here, *S*_*xx*_(*f*_1_, *f*_2_) is the power spectrum of the original image, *S*_*nn*_(*f*_1_, *f*_2_) is the adaptive noise, and *H*(*f*_1_, *f*_2_) is the blurring filter.

### 2.3. 2D-DWT

#### 2.3.1. Advantage of Wavelet Transform

The FT is the most commonly used tool for the analysis of signals, and it breaks down a time-domain signal into constituent sinusoids of various frequencies, thus changing the signal from the time domain to the frequency domain. Nevertheless, the FT has a serious disadvantage as it removes the time information from the signal. For instance, an investigator cannot determine when a specific event took place based on a Fourier spectrum. Therefore, the classification accuracy decreases as the time information is lost.

Gabor modified the FT to examine only a small part of the signal at a time. This approach is known as windowing or the short-time FT (STFT) [23]. It accumulates a window of appropriate shape to the signal. STFT can be considered as a compromise between the time information and frequency information. Nevertheless, the precision of the information is limited by the window size.

**Figure 3:**
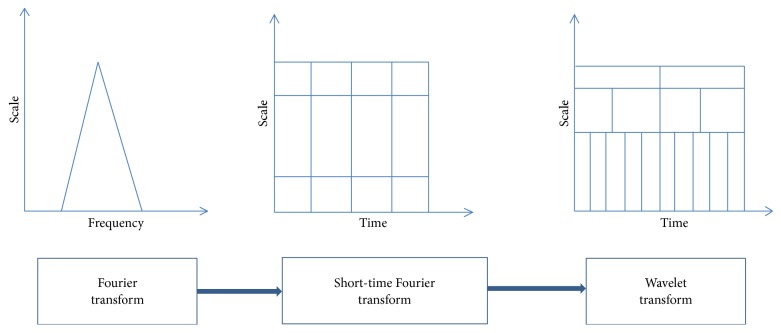
Progress of signal analysis.

The wavelet transform (WT) constitutes the next logical step. It uses a windowing method with variable size, and the progress of the signal analysis is shown in Figure 3. Another benefit of the WT is that it selects a “scale” in place of the traditional “frequency”; that is, it does not generate a time-frequency view of a specific signal but a time-scale view. The time-scale view is another way of visualizing data and is more commonly used and effective.

#### 2.3.2. DWT

This is an effective implementation of the WT, and it utilizes the dyadic scales and positions [24]. The fundamentals of the DWT are as follows. Let *x*(*t*) be a square-integral function. The continuous WT of the signal *x*(*t*) relative to a real-valued wavelet *ψ*(t) is defined as

(2) $$\begin{array}{c} W\left(\;a,\tau \;\right)=\overset{\infty }{\underset{-\infty }{\int }}x\left(\;t\;\right)\frac{1}{\sqrt{a}}\psi *\left(\;\frac{t-\tau }{a}\;\right)dt, \end{array}$$

where *W*(*a*, *τ*) is the WT, *τ* indicates the function across *x*(*t*), and the variable *a* is the dilation factor (both real and positive numbers). Here, the asterisk (*∗*) indicates the complex conjugate.

Equation (1) can be discretized by restraining *a* and *τ* to a discrete lattice (*a* = 2^*j*^ and *τ* = 2^*j*^*k*) to provide the DWT, which is given as follows:

(3) cAj,kn=DS∑nxnlj∗n−2jk,cDj,kn=DS∑nxnhj∗n−2jk.

**Figure 4:**
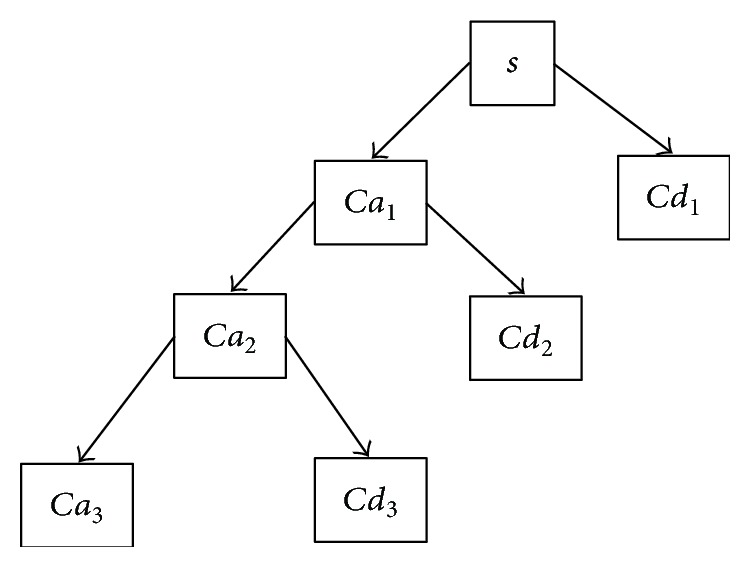
Three-level wavelet decomposition tree.

Here, *cA*_*j*,*k*_ and *cD*_*j*,*k*_ refer to the coefficients of the approximation components and detailed components, respectively. *l*(*n*) and *h*(*n*) represent the LPF and high-pass filter (HPF), respectively. *j* and *k* represent the wavelet scale and translation factors, respectively. The DS operator represents downsampling. The approximation component has low-frequency components of the image, whereas the detailed components contain high-frequency components.  Figure 4 shows a three-level decomposition tree.

#### 2.3.3. 2D-DWT

**Figure 5:**
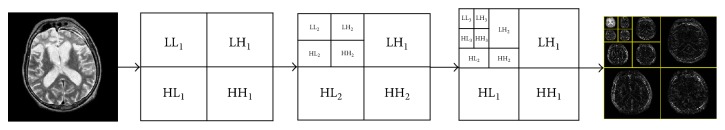
Pathological brain image and its wavelet coefficient at three-level decomposition.

In a case involving 2D images, the DWT is employed in each dimension separately. A sample of a pathological brain MR image with its three-level wavelet decomposition is shown in Figure 5. Consequently, there are four subband images (LL, LH, HH, and HL) at each scale. The subband LL is utilized for the other 2D-DWT and can be considered as the approximation component of the image, whereas the LH, HL, and HH subbands can be considered as the detailed components of the image. As the level of the decomposition is increased, a more compact, but coarser approximation component is accessed. Thus, wavelets give a simple hierarchical foundation for clarifying the image information.

There are various types of wavelets, for example, Daubechies, symlets 1, coiflets 1, and biorthogonal wavelets and reverse biorthogonal 1.1. We tested our result with each type of the wavelet family as shown in Table 2. In our research, the approximation coefficient of three-level wavelet decomposition along with a Haar wavelet yields promising results when compared to others in the wavelet family. Hence, Haar wavelet was selected in the experiment. It is also the simplest and most significant wavelet of the wavelet family. Moreover, it is very fast and can be used to extract basic structural information from an image. All the features are present for all the images, and a feature matrix is generated.

### 2.4. Probabilistic Principal Component Analysis

The PPCA algorithm proposed by Tipping et al. [36–38] is based on the estimation of the principal axes when any input vector has one or more missing values. The PPCA reduces the high-dimensional data to a lower-dimensional representation by relating a *p*-dimensional observation vector *y* to a* k*-dimensional latent (or unobserved) variable *x* that is regarded as normal with zero mean and covariance *I*(*k*). Moreover, PPCA depends on an isotropic error model. The relationship can be established as

(4) $$\begin{array}{c} y^{T}=W*x^{T}+\mu +\varepsilon , \end{array}$$

where *y* denotes the row vector of the observed variable, *ε* denotes the isotropic error term, and *x* is the row vector of latent variables. The error term, *ε*, is Gaussian with zero mean and covariance *v∗I*(*k*), where *v* is the residual variance. To make the residual variance greater than 0, the value of *k* should be smaller than the rank. A standard principal component where *v* equals 0 is the limiting condition of PPCA. The observed variables,* y*, are conditionally independent for the given values of the latent variables *x*. Therefore, the correlation between the observation variables is explained by the latent variables, and the error justifies the variability unique to *y*_*i*_. The dimension of the matrix *W* is *p* × *k*, and it relates both latent and observation variables. The vector *μ* allows the model to acquire a nonzero mean. PPCA considers the values to be missing and arbitrary over the dataset. From this model,

(5) $$\begin{array}{c} y\sim N\left(\;\mu ,W*W^{T}+v*I\left(\;k\;\right)\;\right). \end{array}$$

Given that the solution of *W* and *v* cannot be determined analytically, we used the expectation-maximization (EM) algorithm for the iterative maximization of the corresponding log-likelihood function. The EM algorithm considers missing values as additional latent variables. At convergence, the columns of *W* span the solution subspace. PPCA then yields the orthonormal coefficients.

With respect to our research, the size of the image is 256 × 256. After three-level decomposition, the vector feature becomes 32 × 32 = 1024. Here, all the features are not relevant for the classification. Because of the high computational cost, we utilized PPCA for the dimensionality reduction. The advantage of PPCA over PCA is its computational efficiency.

### 2.5. RSE Classifier

Ensemble classification includes combining multiple classifiers to obtain more accurate predictions than those obtained utilizing individual models. In addition, ensemble learning techniques are considered very useful for upgrading prediction accuracy. Nevertheless, base classifiers must be as precise and diverse as possible to increase the generalization capability of an ensemble model.

For the classification of normal and pathological brain MRI images, we used a random subspace classifier that uses KNN as a base classifier. The main idea behind the success of ensemble classification is the diversification in the classification that makes the ensemble classifier. With the ensemble classification approach, each classifier provides a different error for different instant. Therefore, we can develop a strong classifier that can decrease the error. The random subspace classifier is a machine-learning classifier that divides the entire feature space into subspaces. Each subspace randomly selects features from the original feature space. It must be guaranteed that the boundaries of the particular base classifier are significantly different. To realize this, an unstable or weaker classifier is utilized as base classifier because they create sufficiently varied decision boundaries, even for small disturbances in the training data parameters.

We used the majority voting method to obtain the final decision of the class membership. In the proposed algorithm, we used KNN as the base classifier owing to its simplicity. After selecting a random subspace, a new set of KNNs is estimated. The majority voting method was utilized to combine the output of each base classifier for the decision preparing test class.

### 2.6. Pseudocode of Proposed System

Our proposed system can be outlined in four major stages. The steps involved are depicted in Pseudocode 1.

### 2.7. Performance Measures

Various techniques are used to evaluate the classifier's efficiency. The performance is determined based on the final confusion matrix. The confusion matrix holds correct and incorrect classification results.  Table 1 illustrates a confusion matrix for binary classification, where TP, TN, FP, and FN depict true positive, true negative, false positive, and false negative, respectively.

Here, pathological brains are assumed to hold the value “true,” and normal control (NC) ones are assumed to hold the value “false” following normal convention. Now, we calculate the performance of the proposed approach on the basis of sensitivity, specificity, accuracy, and precision as follows.

(i) Sensitivity (true positive rate): this is the tendency or ability to determine that the diagnostic test is positive when the person has the disease:

(6) $$\begin{array}{c} Sensitivity=\frac{TP}{TP+FN}. \end{array}$$

(ii) Specificity (true negative rate): this is the tendency or ability to determine that the diagnostic test is negative when the person does not have the disease:

(7) $$\begin{array}{c} Specificity=\frac{TN}{TN+FP}. \end{array}$$

(iii) Accuracy: this is a measure of how many diagnostic tests are correctly performed:

(8) $$\begin{array}{c} Accuracy=\frac{TP+TN}{TP+TN+FP+FN}. \end{array}$$

(iv) The precision and the recall are formulated by

(9) $$\begin{array}{c} Precision=\frac{TP}{TP+FP}. \end{array}$$

### 2.8. Cross-Validation

**Figure 6:**
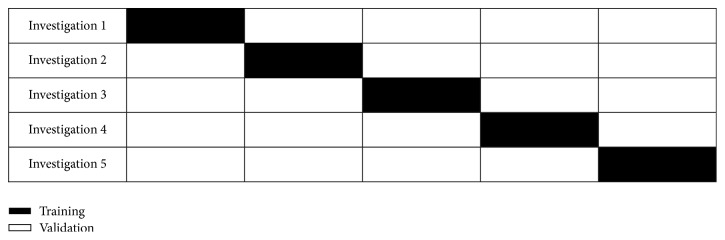
Illustration of *k*-fold cross-validation.

Cross-validation (CV) is a model-assessment method that is used to evaluate the performance of a machine-learning algorithm prediction on a new DS on which it has not been trained. It helps to solve the overfitting problems. Each cross-validation round involves randomly portioning the original DS into a training set and a validation set. The illustration of the *k*-fold CV is shown in Figure 6. The training set is used to train a supervised learning algorithm, while a test set is used to evaluate its performance.

To make the RSE classifier more reliable and generalize to independent datasets, a 5 × 6-fold stratified cross-validation (SCV) and 5 × 5-fold SCV are employed. A 5 × 6-fold SCV is employed for DS-66 and 5 × 5-fold SCV is used for DS-90, DS-160, and DS-255. For DS-66, 55 MR images are used for training whereas 75, 128, and 204 images are used for DS-90, DS-160, and DS-255 respectively. The validation images for DS-66, DS-90, DS-160, and DS-255 are 11, 15, 32, and 51, respectively.

## 3. Results and Discussion

In this study, we implemented a new machine-learning framework using MATLAB 2016a on an Intel computer with a Core-i5 processor and 16 GB RAM running under the Windows 7 operating system. This program can be tested or run on any computer platform where MATLAB is available.

### 3.1. Feature Extraction and Optimum Wavelet

In the proposed system, the three-level 2D-DWT of the Haar wavelet breaks down the input image into 10 subbands, as illustrated in Figure 5. The top left corner of the wavelet coefficient image (Figure 5) represents the approximation coefficients of the three-level decomposition of the image, whose size is only 32 × 32 = 1024. These obtained features are the initial features. The size of these features is still large, and the matrix size needs to be reduced. Now, these reduced features are sent as the input to the PPCA.

### 3.2. Feature Reduction

The use of PPCA as a dimension-reduction tool reduces the feature size to its desired size. Here, we can take the feature as desired. It is better that the desired number of features should at least preserve more than 90% of the variance. However, in this study, we did not take 95% of the variance because it may lead to a higher computational cost. Researchers have considered different numbers of features. In our case, we first used a small number of features, but the accuracy was poor. However, the result with 13 principal components was excellent. Hence, the proposed method uses 13 principal components to earn higher classification accuracy.

### 3.3. Classification Results

The reduced features were sent to the classifier, and the results obtained with the different classifier are promising. From the experiment, it is seen that the proposed method works well for all four DSs using 13 principal components. The performances obtained with logistic regression, quadratic discriminant analysis, KNN, and RSE classifier with KNN as a base classifier are shown in Table 3. From the table, we see that the proposed method outperforms other methods. We utilized a 5-fold CV for DS-90, DS-160, and DS-255, whereas we utilized a 6-fold CV for DS-66. The RSE classifier obtained an accuracy of 100.00%, 100.00%, 100.00%, and 99.20%, with DS-66, DS-90, DS-160, and DS-255, respectively. The result obtained with the cubic SVM is the same as the RSE classifier for the dataset beside DS-66, where it could only achieve 98.50%.

### 3.4. Comparison with Existing Schemes

To further demonstrate the effectiveness of the proposed approach, we compared 21 existing algorithms. The algorithms and their corresponding results are listed in Tables 4 and 5.  Table 4 shows the comparison result with DS-90. It is evident from Table 4 that our proposed method correctly matched all cases with 100% sensitivity, 100% specificity, 100% precision, and 100% accuracy. A comparison of the obtained results shows that our algorithm is superior to the others. This shows the effectiveness of the preprocessing technique combined with features extracted using the WT and PPCA.  Table 4 shows the result of 5 runs of the proposed system.  Table 5 demonstrates the comparison results over the three DSs in terms of the number of features, number of runs, and average accuracy. Here, some of the recent schemes were run 10 times, while others were run five times. From Tables 4 and 5, we see that most of the techniques achieved excellent classification when subjected to DS-66 as it is smaller in size. However, none of the algorithms achieved 100.00% with DS-90 and DS-160 because DS-255 is larger in size and includes more types of diseased brains; therefore, no current CAD system can earn a perfect classification.

Finally, this proposed “DWT + PPCA + RSE” achieved an accuracy of 100% for DS-66, DS-90, and DS-160 and an accuracy of 99.20% for DS-255, which is comparable with other recent studies and greater than the entire algorithm presented in Table 5. The improvement realized by the recommended scheme appears to be marginal compared with other schemes, but we obtained this result based on a careful statistical analysis (five repetitions of *k*-fold CV). Thus, this improvement is reliable and robust.

## 4. Conclusion

This paper proposed a new cascade model of “2D-DWT + PPCA + RSE” for the detection of pathological brains. The experiments validated its effectiveness as it achieved an accuracy of 99.20%. Our contributions lie in three points. First, we introduced the Wiener filter and showed its effectiveness. Besides this we introduced the PPCA and RSE classifier and proved it gives the better performance when compared with other state-of-the-art algorithms. In this work, we transformed the PBD problem to a binary classification task. We presented a novel method that replaced PCA and introduced RSE classifier. The experiment showed the superiority of our methods to existing approaches.

The proposed algorithm can also be employed in other fields, for example, face recognition, breast cancer detection, and fault detection. Moreover, this method has been validated on the publically available datasets which are limited in size. Also, in the selected dataset, the images are collected during the late and middle stage of diseases; however, the images with disease at early stages need to be considered.

In future research, we may consider images from other modalities like MRSI, PET, and CT to increase robustness to our scheme. The proposed method can be validated on a larger clinical dataset utilizing modern machine-learning techniques like deep learning, extreme learning, and so on, after collecting the enough brain images from the medical institutes. Internet of things can be another promising research field to embed this PBDS.

## Figures and Tables

**Pseudocode 1:**
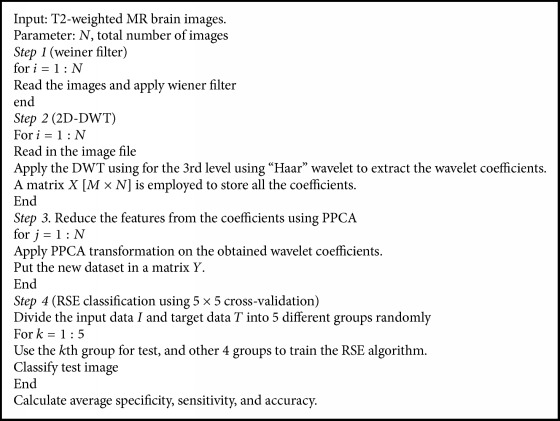
Pseudocode of the proposed system.

**Table 1:** Confusion matrix for a binary classifier to discriminate between two classes (*A*_1_ and *A*_2_).

True class	Predicted class	
	*A*_1_ (patients)	*A*_2_ (controls)
*A*_1_ (patients)	TP	FN
*A*_2_ (controls)	FP	TN

Here, TP (true positive): correctly categorized as positive cases, TN (true negative): correctly categorized as negative cases, FP (false positive): incorrectly categorized as negative cases, FN (false negative): incorrectly categorized as positive cases.

**Table 2:** Comparison of different wavelet families.

Wavelet family	Accuracy
Haar	99.20%
Daubechies 2	98.60%
Coiflets 1	96.98%
Symlets 1	99.01%
Biorthogonal 1.1	98.64%

**Table 3:** Comparison result of the proposed method.

Proposed method	Feature	DS-66	DS-90	DS-160	DS- 255
Logistic regression	13	100.00	100.00	100.00	92.50
QDA	13	100.00	98.90	98.90	96.50
KNN	13	100.00	100.00	100.00	97.30
RSE classifier	13	100.00	100.00	100.00	99.20

**Table 4:** Classification comparison with DS-90.

Existing methods	Success cases	Sensitivity (%)	Specificity (%)	Precision (%)	Accuracy (%)
DWT + PCA + BPNN [25]	388	88.00	56.00	97.14	86.22
DWT + PCA + RBF-NN [25]	411	92.47	72.00	98.25	91.33
DWT + PCA + PSO-KSVM [25]	440	98.12	92.00	99.52	97.78
WE + BPNN [26]	390	88.47	56.00	97.16	86.67
WE + KSVM [27]	413	93.18	68.00	98.02	91.78
DWT + PCA + GA-KSVM [28]	439	97.88	92.00	99.52	97.56
WE + PSO-KSVM [29]	437	97.65	88.00	99.28	97.11
WE + BBO-KSVM [29]	440	98.12	92.00	99.52	97.78
WE + QPSO-KSVM [30]	442	98.59	92.00	99.52	98.22
WFRFT + PCA + GEPSVM [31]	446	99.53	92.00	99.53	99.11
HMI + SEPSVM [15]	445	99.06	96.00		98.89
HMI + TSVM [15]	445	99.29	92.00		98.89
*Proposed*					
2D- DWT + PPCA + RSE (proposed)	450	100.00	100.00	100.00	100.00

**Table 5:** Classification comparison (DS-66, DS-160, and DS-255).

Approaches	Feature	Run	Accuracy (%)		
DWT + SVM + POLY [24]	4761		*DS-66*	*DS-160*	*DS-255*
DWT + SVM + RBF [24]	4761	5	98.00	97.15	96.37
DWT + PCA + *k*-NN [4]	7	5	98.00	97.33	96.18
DWT + PCA + FNN + ACPSO [32]	19	5	98.00	97.54	96.79
DWT + PCA + FNN + SCABC [33]	19	5	100.00	98.75	97.38
DWT + PCA + BPNN + SCG [7]	19	5	100.00	98.93	97.81
DWT + PCA + KSVM [5]	19	5	100.00	98.29	97.14
RT + PCA + LS-SVM [34]	9	5	100.00	99.38	98.82
SWT + PCA + IABAP-FNN [11]	7	10	100.00	98.88	98.43
WT + PCA + ABC-SPSO-FNN [11]	7	10	100.00	99.44	99.18
WE + NBC [35]	7	10	92.58	99.62	99.02
DWT + PCA + ADBRF [17]	13	5	100.00	99.30	98.44
DWT + SUR + ADBSVM [18]	7	5	100.00	99.22	98.43
FRFE + DP-MLP + ARCBBO [16]	12	10	100.00	99.19	98.24
FRFE + BDP-MLP + ARCBBO [16]	12	10	100.00	99.31	98.12
DWT + PCA + RSE	13	5	100.00	99.57	98.90
DWT + PPCA + RSE (proposed)	13	5	100.00	100.00	99.20

## Nomenclature

MR(I): Magnetic resonance (imaging)
DWT: Discrete wavelet transform
PPCA: Probabilistic principal component analysis
KNN: *k*-nearest neighbor
CV: Cross-validation
BPNN: Backpropagation neural network
KSVM: Kernel support vector machine
SCG: Scale conjugate gradient
LS-SVM: Least-square support vector machine
FBPNN: Feedforward backpropagation neural network
SWT: Stationary wavelet transform
PSO: Particle swarm optimization
CAD: Computer-aided diagnosis
STFT: Short-time Fourier transform
QDA: Quadratic discriminant analysis
SUR: Symmetric uncertainty ranking
PZM: Pseudo Zernike moment
SWT: Stationary wavelet transform
DTCWT: Dual-tree complex wavelet transform
RBFNN: Radial basis function neural network
CT: Computed tomography
TSVM: Twin support vector machine
HMI: Hu moment invariants
MLP: Multilayer perceptron
ARCBBO: Adaptive real coded biogeography-based optimization
DP: Dynamic pruning.
